# Beyond forced translation: obesity, disability and the case for autonomous protection against weight discrimination

**DOI:** 10.3389/fpubh.2026.1937485

**Published:** 2026-09-22

**Authors:** Raúl Sampieri-Cabrera, Karen Soldatic

**Affiliations:** 1 Department of Physiology, Faculty of Medicine, National Autonomous University of Mexico, Mexico, Mexico; 2 Centro de Ciencias de la Complejidad, Universidad Nacional Autónoma de México, Ciudad de México, Mexico; 3 The CERC in Health Equity and Community Wellbeing, Toronto Metropolitan University, Toronto, ON, Canada

**Keywords:** disability, human rights, obesity, public policies, weight bias, weight discrimination

## Abstract

Legal and public health debates on obesity move between three distinct objects: a clinical condition, a functional limitation, and a process of social exclusion. Explicit protection against discrimination based on body weight remains exceptional across jurisdictions, and legal systems in different countries have turned to disability law as the available route for people with obesity who face exclusion. This perspective argues that protection against weight-based discrimination should not depend on prior demonstration of disability. Where an explicit ground is absent, people harmed by weight discrimination must translate their claim into the language of disability, and that translation reaches only the portion of the harm expressible as lasting functional limitation. The Obesity–Disability–Discrimination Triangle is proposed as an analytical framework that maps the configurations in which the three phenomena combine and assigns the normative response required for each. Although disability framing brings substantial benefits in terms of justiciability and clinical orientation toward actual functioning, it also introduces serious risks. Among them are the overmedicalization of corporeality, the reinforcement of paternalistic stigmas of “incapacity” and a legal substitution effect that makes invisible the discriminatory violence exercised against people with large bodies who do not have any functional restriction. The ideal ethical and legal solution must be hybrid. Legal systems should maintain disability protection for cases with real and lasting functional limitations. However, they should urgently institute weight discrimination as a category of autonomous exclusion in employment, education, and health, protecting bodily diversity as a fundamental human right, independent of health or ability.

## Introduction

There is a broad consensus on recognizing obesity as a chronic and relapsing disease (1–3). Within this broad clinical spectrum, some manifestations and complications generate persistent functional limitations that can be framed within the legal framework of disability (4, 5). At the same time, a considerable proportion of the disadvantages experienced by people with obesity are related to processes of stigmatization, discrimination, and social exclusion rather than to functional restrictions themselves (6, 7).

The World Health Organization characterizes obesity as a chronic and relapsing disease resulting from the interaction between biological, behavioral, environmental, and socioeconomic factors, including genetics, neurobiology, dietary patterns, access to healthy food, and food market dynamics (8). Their estimates indicate that in 2022, approximately 890 million adults lived with obesity and that the global prevalence in the adult population had more than doubled since 1990 (9).

The dimension of obesity in public health is so high that it has reoriented the legal debate, where the discussion revolves around the regulatory instruments capable of offering effective protection against forms of exclusion associated with body weight discrimination (10–12). The World Obesity Federation identifies weight stigma as both a social determinant of health and a human rights issue (13). Consistently, the International Consensus Statement on Obesity Stigma published in 2020 concluded that these forms of discrimination generate physical and psychological harm and limit access to adequate health care (14).

Explicit legal protection against weight discrimination is exceptional. Most jurisdictions omit body weight and physical appearance from the list of prohibited grounds, and the protections that do exist are largely subnational, as with the Elliott-Larsen Civil Rights Act in Michigan and the physical features recognized in the State of Victoria in Australia (11, 15, 16). Disability law has operated as the available substitute within that vacuum, and people excluded from employment, education, health care, or services have obtained remedies by arguing that their weight constitutes an impairment, that it produces lasting functional limitations, or that they were treated as though it did (17–19). The volume of harm routed through this single channel is considerable. A meta-analysis of observational studies estimated the pooled prevalence of perceived weight discrimination at 19.2% (95% CI 11.7 to 29.8) among adults with class I obesity and at 41.8% (95% CI 36.9 to 46.9) among adults with a body mass index above 35 kg/m^2^, with high heterogeneity in both pooled estimates (I^2^ above 95%) to warrant reading these figures as orders of magnitude and not as precise point estimates (20). Reported discrimination concentrates in employment, health care, education, and everyday interpersonal settings, rises with body mass index, and is more frequent among women (Figure 1).

**Figure 1:**
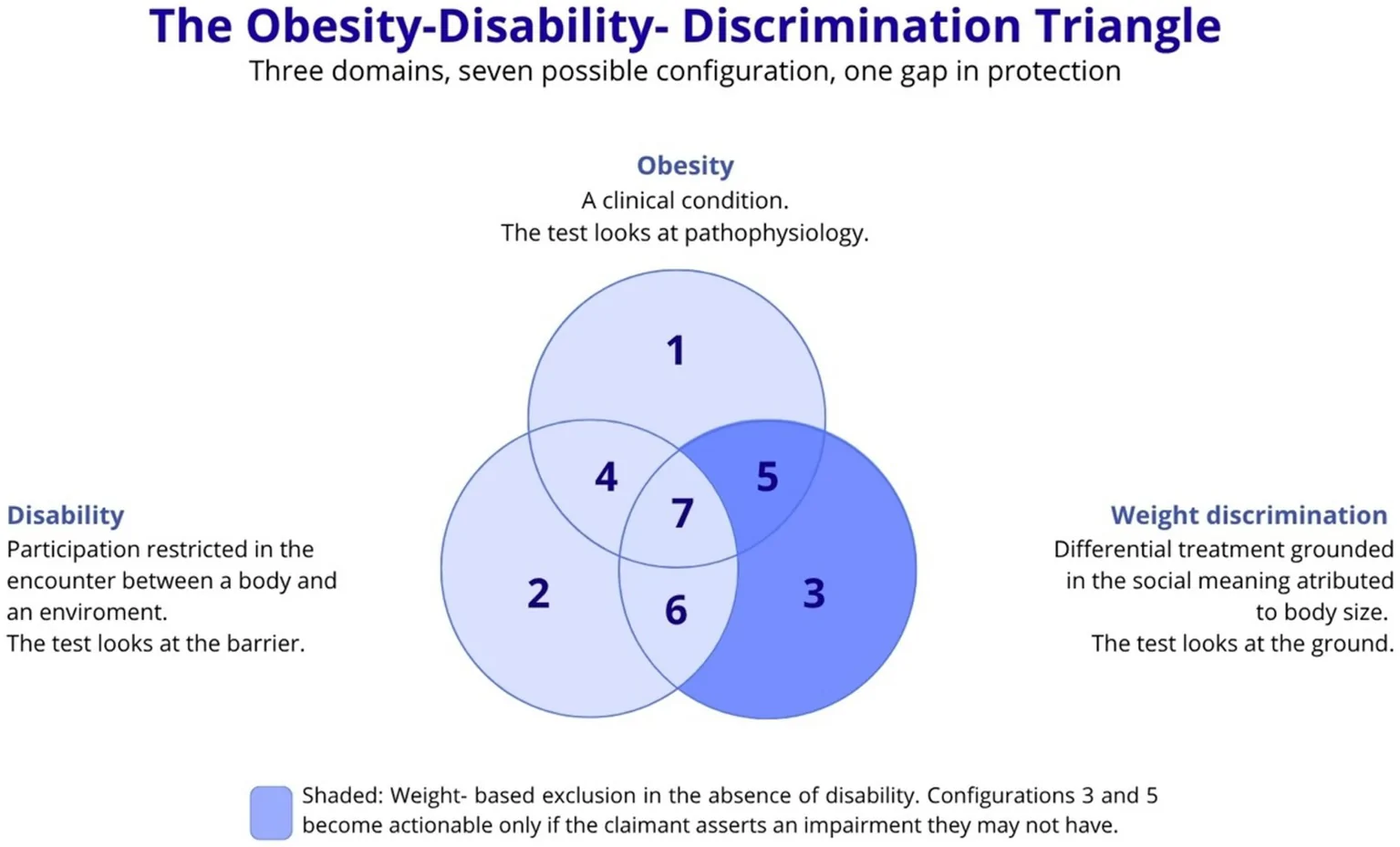
The Obesity–Disability–Discrimination Triangle: Obesity, disability and weight-based discrimination as distinct but intersecting normative domains. Each domain is established by a different test. Obesity is established by reference to pathophysiology, disability by reference to the barrier that restricts participation, and weight discrimination by reference to the ground of the differential treatment. Because the three tests are independent, a single person may satisfy one, two or all three at once, and the above three domains therefore combine in seven configurations, numbered in the figure and specified in Table 1. Belong area covers configurations 3 and 5, in which weight-based exclusion occurs in the absence of disability. Those two configurations are the analytic core of the argument developed here, since they become actionable under disability law only if the claimant asserts an impairment they may not have.

Canada displays the consequences of this arrangement with particular clarity. The *Canadian Human Rights Act* lists disability among prohibited grounds and omits body weight (21), and the Ontario Human Rights Commission records that claims involving weight have advanced through actual or perceived disability (22). An environmental scan of Canadian case law concludes that current disability protections are inadequate for people who experience weight discrimination without experiencing disability and argues that weight should be recognized as a human rights issue in its own right (23). The problem addressed in this study is evident from this conclusion. The difficulty lies less in an assertion by courts or scholars that obesity and disability are identical and more in the fact that the absence of an autonomous ground obliges claimants to render an experience of weight-based exclusion in the vocabulary of functional impairment, which leaves unremedied whatever portion of the harm resists that rendering (Table 1).

**Table 1:** The seven configurations generated by the Obesity–Disability–Discrimination Triangle, the normative response owed in each, and whether disability law alone reaches the harm.

No.	Configuration	Normative response owed	Reached under disability law alone
1	Obesity without restricted participation or weight-based exclusion	Clinical care	Not applicable, since no discrimination claim arises
2	Disability without obesity or weight-based exclusion	Accommodation and environmental redesign	Yes
3	Weight-based exclusion without obesity or disability	Prohibition of differential treatment	No
4	Obesity with participation restricted by environmental barriers	Care and accommodation	Yes
5	Obesity with weight-based exclusion and participation intact	Care and prohibition of differential treatment	No
6	Disability with concurrent weight-based exclusion	Accommodation and prohibition of differential treatment	In part only, and only through impairment
7	Obesity, restricted participation and weight-based exclusion concurrently	Care, accommodation and prohibition of differential treatment	In part only, and only through impairment

Configurations 3 and 5 correspond to the shaded area of Figure 1.

Shading marks the two configurations in which weight-based exclusion occurs in the absence of a disability. The boundary between accommodation and prohibition follows the normative response owed and not the causal location of the harm, since environments designed around a normative range of bodies restrict participation and simultaneously express devaluation at the same time. Clinical obesity, defined by alterations in activities of daily living, falls into configurations 4 and 7 whenever an environment converts the limitation into restricted participation.

From a comparative perspective, the use of disability law to protect people with obesity has often been a pragmatic response to regulatory gaps (17, 19). However, its scope is limited when it becomes the main mechanism for addressing discrimination based on weight (23). The discussion is more than terminological or technical, since it defines which experiences of damage acquire legal recognition, which can be subject to reparation, and which remain outside the field of legal protection because they do not correspond to a strict notion of functional limitation. This interpretation arises from the convergence of biomedical evidence, the conceptual frameworks of disability promoted by the WHO, and the diversity of legal trajectories observed in the European Union, the United States, Canada, the United Kingdom, and Australia.

This article addresses the resulting normative architecture. What is at stake is distributive and expressive at once, since the categories a legal system recognizes determine which injuries can be named, remedied, and deterred, and which remain outside the perimeter of protection. Our purpose is to specify the relations among obesity, disability, and weight discrimination with sufficient precision to support a legislative agenda in which weight operates as an autonomous protected ground while disability protection remains available wherever lasting functional limitation exists. The Obesity–Disability–Discrimination Triangle is the instrument offered for this specification. Critical scholarship on fatness and law has already established that the three phenomena are distinguishable and debated the political costs of routing weight claims through disability (15, 16, 24–27). The framework proposed here builds on that work by mapping the configurations in which the three phenomena combine and by assigning to each configuration the normative response it requires, which allows the case for an autonomous ground to be stated in terms that legislators and adjudicators can operationalize. We defend the implication of a hybrid design that retains disability protection where functional limitation is real and lasting and adds weight discrimination as an independent prohibited ground in employment, education, health care, and services.

## Why obesity, disability and discrimination are not the same phenomenon

Three distinctions organise this section. The first is biomedical and concerns what obesity is named as a clinical object. The second is functional and relational and concerns the conditions under which disabilities arise. The third is socio-legal and concerns the operation of weight discrimination, independent of clinical status or functional capacity. Each distinction rests on a different body of authority and licenses a different normative response, which is when the three collapse into one another, it is at a cost.

The first distinction is biomedical: “Obesity” names a bodily and metabolic condition (28); it does not name a univocal pattern of incapacity. The WHO characterizes it as a chronic disease, and a recent commission published in *The Lancet Diabetes & Endocrinology* defined “clinical obesity” as a chronic systemic disease marked by alterations in the function of tissues, organs, the entire individual, or activities of daily living (ADL) (29–31). This shift is important, even within obesity medicine, since the discussion is no longer limited to excess adiposity as a bodily trait but also includes its functional and organic consequences.

The reference to activities of daily living brings clinical obesity into proximity with functional definitions of disability and requires an explicit account of the boundaries. The proximity is real, and the framework proposed here treats it as an expected overlap and not as an anomaly. Clinical obesity and disability differ in the operations performed in each category. Clinical obesity is a diagnostic judgement that attributes an observed alteration in tissue, organ, or whole-person function to excess adiposity, thereby grounding a duty of care, with the reference point located in pathophysiology and the attribution running from adiposity to impairment. Disability is a relational and legal judgement about restricted participation produced in the encounter between a person and an environment, and it grounds duties of accommodation and non-discrimination, with the reference point located in the barrier. A person with clinical obesity who cannot climb a flight of stairs because of exertional dyspnea satisfies the first judgement once the limitation is attributed to adiposity, and satisfies the second only where the built environment, workplace, or service in question converts that limitation into exclusion from participation. Therefore, the two categories pick out overlapping populations while remaining analytically distinct, and neither can be derived from the other. The consequence of the argument developed here is that a diagnosis of clinical obesity carries no automatic entitlement to disability status and no automatic exclusion from it, and that neither determination settles whether the person has been discriminated against on the basis of body weight.

The second distinction is functional and relational: for the WHO, disability results from the interaction between a health condition and personal and environmental factors (see UNCRPD Art. 1). The WHO insists that the environment decisively shapes the experience and extent of disability (32). The United Nations Convention on the Rights of Persons with Disabilities (UNCRPD) (33), which provides the primary international human rights framework for disability, establishes that disability emerges through the interaction between persons with impairments and attitudinal and environmental barriers that hinder their full and effective participation in society on an equal basis. Consistent with this approach, the International Classification of Functioning, Disability and Health (ICF) (34) conceptualizes disability as a multidimensional and contextual phenomenon involving dynamic interactions between body functions, activities, participation, and environmental factors.

The third distinction is socio-legal; weight discrimination can exist even in the absence of a disability (35, 36). Perceived weight discrimination is reported by a substantial minority of adults with obesity, and its frequency increases with body mass index (20). The World Obesity Federation defines weight stigma as a set of stereotypes, prejudices, and structures that harm health and equity of care. The 2020 International Consensus Declaration and recent reviews show that weight stigma manifests itself in healthcare, employment, education, and daily life, and that it results in barriers to access, poorer clinical-patient communication, avoidance of services, and psychological harm (13).

This analytical separation has important consequences for people; for example, one person may live with obesity without presenting a relevant functional limitation, another may live with obesity and have substantial limitations in mobility, sleep, breathing, or pain, and a third may not have any functional limitation and, even so, suffer exclusion from school, work, or health due to the social meaning attributed to their body size. Mixing these three levels (illness, disability, and discrimination) produces different normative errors: either the disability is overdiagnosed, the real disability is denied, or the discriminatory violence that operates even in the absence of disability is erased. In its 2025 fact sheet, the WHO distinguishes between obesity treatment and the assessment of comorbidities and disabilities. Simultaneously, the WHO insists that stigma and discrimination are mechanisms that produce functional and social inequality (8).

## Comparative legal trajectories

This section traces how the five legal systems have handled claims arising from body weight. The comparison is organized around what a claimant must establish before a legal system will hear the claim. The answer converges across jurisdictions on lasting functional limitations, whether formulated as impairment, substantial effect on major life activities, or hindrance to full and effective participation in professional life. This convergence supplies the empirical basis for the argument that follows, since it shows that the translation requirement is a structural feature of the available legal instruments and not an accident of any single jurisdiction.

In the European Union, Directive 2000/78/EC (37) establishes a general framework for equality in employment and occupation and requires reasonable accommodation for people with disabilities. In the line opened by *Kaltoft v Municipality of Billund* (38), obesity was not recognized as a protected ground *per se*; what remained open was that obesity could constitute a disability when it generated lasting physical, mental, or psychological limitations that hindered full and effective participation in professional life. The dominant assumption in Europe, therefore, is not “obesity = disability,” but “obesity + lasting functional limitation = possible disability” within anti-discrimination labor law.

In the United States, there is a broad legal design in the abstract, but it is restrictive in judicial practice. The *Americans with Disabilities Act* (ADA), substantially amended by the *ADA Amendments Act* (ADAAA) of 2008, mandates an extremely lax interpretation of protective coverage, explicitly recognizing the impact of impairments on “major life activities” and “major bodily functions” (U.S. Congress, 2008) (39). Weight is not among the bases of discrimination prohibited by federal statutes enforced by the U.S. Equal Employment Opportunity Commission (39). For years, the EEOC actively litigated an expansive reading in which it held that severe or morbid obesity represented an intrinsic physiological impediment that did not require proof of an underlying pathology, achieving favorable rulings in trial courts (40, 41). Despite this, federal jurisprudence from the Courts of Appeals has firmly determined that obesity is not an impairment under the ADA unless it is a documented result of an underlying physiological disorder (42). This interpretive divergence between the directives of federal agencies and the restrictive decisions of the judicial circuits confirms that, while the United States conceptual framework is broad, the legal status of obesity remains doctrinally unstable.

On the other hand, the Washington State Supreme Court held in *Taylor v. Burlington Northern Railroad Holdings* that obesity is an impairment covered by the Washington Law against Discrimination and expressly noted that it is illegal to refuse to hire a person living with obesity if they are otherwise qualified for the job. In addition, the EEOC recognizes that several subnational entities have laws that specifically prohibit height and weight discrimination unless there is an actual job requirement. This is important because it demonstrates that an institutional alternative exists, as institutional anti-discrimination frameworks recognize that obesity or body size is not a disability in itself, thereby providing a distinct legal basis for protection rather than requiring all weight-based exclusion to be addressed through disability law (43).

In Canada, the *Canadian Human Rights Act* lists disability as a prohibited ground but does not include body weight as an autonomous category of discrimination. The Ontario Human Rights Commission recognizes that historical jurisprudence tended to treat obesity as a disability only if it resulted from an injury, a birth defect, or a disease. Simultaneously, it points out that more recent decisions accepted that extreme obesity can be a disability and that there is also protection when the person is perceived as disabled because of their obesity or because of limitations attributed to it. A 2025 study on the legality of weight discrimination in Canada concludes that, precisely because weight is not explicitly protected, people must argue their cases by way of disability; that same paper considers that solution insufficient and argues that weight should be a bona fide human rights issue, independent of disability protections. Canada is perhaps the clearest example of a system that uses disability law as a substitute for more targeted anti-discrimination protection (21).

In the United Kingdom, the structure is similar to the European one but is more explicit in its interpretive technique. The *Equality Act 2010* protects disability as a protected characteristic but not weight as a protected characteristic. The official government guidance offers an example that is almost a condensed legal thesis: obesity itself is not an alteration, but it causes breathing and mobility difficulties that substantially affect the ability to walk. The guidance also notes that the effect of the alteration is important, not its cause. Subsequent case law cited *by Walker v. SITA* reiterated the same principle: obesity is not, in and of itself, a disability, but it may make it more likely that there is a disability arising from other impairments or their cumulative effects (44).

In Australia, the *Disability Discrimination Act 1992* defines disability in terms that include total or partial loss of bodily or mental functions, loss of body parts, malformation, malfunction, or disfigurement, and also covers present, past, future, or imputed conditions. The law prohibits discrimination in employment, education, access to real estate, goods and services, and other areas. However, the Australian Human Rights Commission has maintained that obesity cases are decided both ways and that the issue requires a case-by-case approach to be effective. When weight does impair functioning, or when the person is treated as if they were impaired, there is coverage; apart from that, framing is not automatic. Australia also offers a relevant track for the future; in Victoria, discrimination by physical features expressly protects attributes such as height, weight, size, and shape. This solution avoids forcing any weight claims into the disability mold (45).

A comparison of the different regulatory frameworks of the countries reveals that the law is willing to protect against obesity when it can be translated into functional disability. However, it is much less consistent when the harm is weight exclusion in the absence of disability. This is the most important conceptual flaw in the current debate on the topic.

## Pragmatic benefits of disability framing

Recognizing obesity within disability frameworks has real advantages. First, it makes part of the damage justiciable and opens the door to actions for discrimination, reasonable accommodations, and reviews of labor or institutional decisions based on stereotypes. In the EU, Directive 2000/78 links disability with reasonable accommodation; in the United States, the ADA requires reasonable accommodations except for due hardship; in Australia, the DDA is deployed in employment, education, and services; and in the United Kingdom, the official logic focuses on the functional effect and not on the cause. For people living with severe obesity, whose limitations are real and long-lasting, this is not a minor advantage; without this doctrinal bridge, many would be outside the perimeter of protection.

The second advantage is epistemic and clinical: disability law forces us to shift the question from the moral judgment on weight to the analysis of functioning, participation, and barriers. This reorientation is consistent with the WHO’s International Classification of Functioning, Disability, and Health, which is effectively linked to the UN Convention on the Rights of Persons with Disabilities (2006) and the relational definition of disability adopted by the WHO. The reorientation converges, at least partially, with the recent shift in clinical obesity towards finer functional and organic criteria. Thus, the disability pathway not only protects people living with obesity but also corrects the societal tendency to trivialize the functional suffering associated with certain forms of severe obesity (46).

The third advantage is its strategic approach. In jurisdictions where weight is not a protected category, disability is the only vehicle available to stop overtly exclusionary decisions from being made. In Canada, the OHRC recognizes this evolution, and the 2025 study documents precisely that, in the absence of explicit weight protection (47), claims are made through actual or perceived disability. Such a strategy may be conceptually imperfect, but normatively, it has made it possible to obtain concrete victories where otherwise there would be no protection at all (22).

## Conceptual risks and unintended effects

However, a useful solution is not necessarily adequate. The first risk is over-medicalization. If all obesity is rewritten as a disability, the law confuses adiposity, disease, and social participation (13). This clashes with the core of the WHO and ICF relational approach to disability, according to which disability does not reside exclusively in the body but in the interaction between health conditions and environmental barriers (48, 49). When the legal category of disability is used to cover any inequality by weight without discriminating between cases, it loses the capacity to separate configurations that call for accommodation and environmental redesign from those that call for the prohibition of differential treatment.

This distinction does not place limitations inside bodies or exclusion inside environments. Social and relational scholarship has contested this division for decades, and work at the intersection of fat studies and disability studies has shown that the two operate together (24–27). A larger-bodied person who cannot use fixed seating, a diagnostic scanner, an aircraft cabin, or a clinical examination table encounters a limitation produced by the interaction between a body and environments designed around a normative range of bodies, and the same design decisions communicate that bodies outside that range were not anticipated. Limitation and devaluation are jointly present in such cases, and any framework that assigns them to separate causal compartments will misdescribe them. The distinction we draw runs along the normative response owed and not along the causal location of harm. Where participation depends on the material configuration of an environment, the appropriate response is accommodation, redesign, and universal accessibility, and disability law supplies the doctrinal machinery for it. Where participation depends on the social meaning attributed to body size, the appropriate response is the prohibition of differential treatment on that ground, and disability law reaches it only through the detour of impairment. Configurations in which both responses are owed are common, and the framework is built to make this concurrence visible instead of forcing a choice between the two. The objection to routing every weight claim through disability is therefore not that fat embodiment falls outside the relational model, which plainly accommodates it, but that the doctrinal price of entry is an assertion of impairment that many claimants have no reason to make and that carries its own (16).

The second risk is the reinforcement of stigma; the weight stigma literature shows that heavier people already face stereotypes of laziness, lack of self-control, lower intelligence, or incompetence, with documented effects on healthcare and employment (50, 51). If we add to these stereotypes a social reading of “incapacity” applied indiscriminately, the result may not be liberating but paternalistic, with more formal tutelage, but also lower expectations, subtle segregation, and protective institutional decisions that actually restrict opportunities. Direct evidence on whether the legal label of disability reduces or increases weight stigma remains scarce. What we do know is that weight stigma is structural, that it is present among health professionals and students, and that educational interventions only modestly reduce explicit bias rather than implicit bias (18, 52). Therefore, it would be naïve to suppose that a legal reclassification will resolve the cultural regime of corporal contempt.

The third risk is what could be called the legal substitution effect. When the law lacks an explicit avenue against weight discrimination, disability absorbs claims that conceptually do not belong to it. The study by (23) states this with remarkable clarity: current disability protections are inadequate for those who suffer from weight discrimination but do not experience a disability because of their weight. This observation transcends Canada (53). This also explains the European (54, 55), British (56), and Australian patterns (57): the law protects when there is a functional limitation, but it does not know what to do when the problem is that a large body is considered socially undesirable, inappropriate, or less valuable even if that body (58, 59).

In addition, another risk is distributional, since welfare, employment, and health systems operate with specific thresholds and categories. If obesity is classified as a disability without robust functional criteria, controversies regarding eligibility, accommodation, and inherent job requirements will increase. The Australian response is instructive; the AHRC suggests a case-by-case analysis focused on the functioning and imputation of disability. This caution reflects correct intuition, since the problem is not the moral expansion of the circle of protection but the loss of conceptual precision in the allocation of rights, duties, and resources (60).

## The obesity–disability–discrimination triangle

We propose an analytical framework that distinguishes obesity, disability, and weight discrimination as related but non-equivalent phenomena. The need for a framework arises from a specific and consequential state of affairs. Where no statute prohibits discrimination based on body weight, a person living with obesity who has no functional limitations and is refused employment, graded down, denied a service, or humiliated in a clinical encounter holds no ground on which to bring a claim, and the only available route requires that person to assert an impairment they do not have or to accept that the injury is not legally cognizable. Medical, functional, and legal categories are treated as interchangeable in public and specialized debates, and this interchangeability sustains the arrangement by making the missing ground appear redundant. Separating the categories shows that the ground is missing and that the harm it would address is not covered elsewhere.

Obesity refers, in the first place, to a clinical condition characterized by an excessive accumulation of adipose tissue associated with physiological alterations, health risks and possible disease trajectories (3, 61, 62). From this perspective, the main concerns are prevention, treatment, and healthcare (63–65). The biomedical and clinical literature has widely consolidated this interpretation and incorporated it into the conceptual framework of chronic diseases (66–68).

Disability introduces a new dimension. What is relevant is no longer the diagnosis or the magnitude of adiposity but the existence of substantial restrictions on activity or social participation (69). Such constraints cannot be understood solely based on individual bodily characteristics, as they emerge from the interaction between these characteristics and the material, institutional, and social conditions of the environment (70). This understanding is reflected in the International Classification of Functioning, Disability, and Health (ICF) and represents one of the pillars of disability models (71–73).

Weight discrimination operates within a framework independent of the presence of disease or functional limitations. Its etiology lies in the processes of social stigmatization that moralize body anthropometry, generating tangible disparities in the work, educational, health, and daily environments (74, 75). Evidence accumulated in recent decades shows that these dynamics of exclusion have a measurable adverse impact on comprehensive health (52) (physical and mental) and restrict the socioeconomic development of the affected individuals (50).

The relationship between these three categories is not fixed or linear, and some people living with obesity develop functional limitations that can acquire legal relevance under disability frameworks. Others experience weight-related discrimination without significant functional limitations or health problems. There are also situations in which functional limitations and stigmatization processes overlap and reinforce each other. Recognizing these possibilities avoids automatically identifying obesity as a disability and reduces the weight-related experiences of exclusion to an exclusively medical problem. The main analytical virtue of the model is to preserve these differences and allow each situation to be evaluated through the conceptual and normative categories that best describe its nature.

Previous studies have established the separation on conceptual and political grounds and debated whether fatness should be claimed as a disability at all (15, 16, 24–27). The addition made here is operational in nature. Assigning a distinct normative response to each configuration converts a conceptual claim into a criterion for legislative and adjudicative design, as it allows a legal system to determine whether a given exclusion calls for accommodation, the prohibition of differential treatment, or both, without requiring the claimant to establish disability as a precondition for either. The framework also identifies the point at which reform is needed. Jurisdictions that already protect physical features or body size, as the State of Victoria does, address the third configuration directly, while jurisdictions that rely on disability alone address it only when the claimant can also satisfy the second, which this article argues should end.

## Implications for health, employment and public policy

The International Classification of Functioning, Disability, and Health is a way to separate issues that are usually mixed under the diagnosis of obesity. A clinical diagnosis provides information about adiposity, comorbidities, and health risks but says little about a person’s degree of social participation or the barriers they face in their daily lives (69, 73). Therefore, the WHO (34) recommends specifically assessing the functional limitations associated with each case of MSD. In addition, there is evidence that identifies weight-related stigma as a factor that deteriorates the quality of care, reduces the use of services, and affects the therapeutic relationship (76, 77). Educational interventions aimed at professionals seem to partially diminish certain forms of explicit bias, although the changes observed in implicit biases remain modest and heterogeneous (78).

Labor controversies show the limits of classification based solely on body size. Legal conflicts rarely revolve around weight itself; they tend to focus on performance, security, access to employment, or permanence in the workplace (23). Both recent jurisprudence and various regulatory orientations converge in that the evaluation should focus on effective capabilities, specific requirements of the position, and possible reasonable accommodations (79, 80). Legal relevance arises from the relationship between a person and the tasks they perform, not from an anthropometric value considered in isolation.

Among children and adolescents, the principal harms follow from weight stigma and discrimination rather than from body weight itself, and they appear in social relationships through ridicule, exclusion, and weight-based victimization that affect psychological well-being, academic performance, and school participation (53). Weight-based victimization is among the most frequently reported forms of peer harassment among young people with high body weight. It originates with peers, teachers, and family members alike, and the documented sequelae include depressive symptoms, disordered eating, avoidance of physical activity, and disengagement from school, all of which follow from the treatment received and not from adiposity (81). UNICEF has pointed out that a large part of the psychosocial consequences associated with obesity is mediated by stigmatization processes (82). Interestingly, many anti-bullying policies continue to pay limited attention to this specific form of victimization despite consistent evidence (83).

International regulatory experience shows a progressive differentiation between functional limitations and discrimination based on bodily appearance. Some jurisdictions continue to address these conflicts solely through disability categories, while others have incorporated specific protections related to weight, height, and physical characteristics (84). That is, exclusion produced by aesthetic prejudices, moral stereotypes, or social expectations about the body does not always find an adequate description in the legal language of disability, even when its material consequences may be equally significant (15, 36).

Public health discussions have begun to recognize that stigma represents a structural component of the experience of many people living with obesity (50, 67). International organizations and scientific associations increasingly agree that social, economic, and cultural environments actively participate in the production of problems (85, 86). From this perspective, campaigns built on blame, shame, or body surveillance run the risk of reinforcing mechanisms that hinder prevention and treatment (87, 88). Trust in healthcare institutions, willingness to seek help, and therapeutic adherence depend, in part, on individuals not anticipating experiences of humiliation or degrading treatment (6, 89).

However, empirical questions persist that still lack sufficiently robust answers, such as: Little is known about the long-term effects of recognizing obesity under legal frameworks for disability, and there is no conclusive evidence of its impact on social attitudes or specific experiences of exclusion. The interplay between body weight, sex, socioeconomic status, race, and pre-existing disability remains a relatively fragmented field (25, 90, 91). A similar situation occurs in the evaluation of laws specifically aimed at combating weight discrimination. The dialogue between obesity research and disability studies could contribute to closing some of these gaps by integrating clinical knowledge, functional analysis, and the study of social barriers within the same analytical framework.

### Conclusion and open-ended questions

Obesity should not be conceptualized or regulated as a disability. This comparison is too crude for the complexity of the phenomenon. Obesity can be a disease; it can, in some cases, cause disability and trigger discrimination even when it does not produce disability. Treating these three levels as equivalent is useful for litigating some cases, but is detrimental for thinking inclusively, legislating fairly, and designing effective public policies.

Therefore, the most appropriate legal and ethical mechanism for protecting people against weight-related exclusion should be a hybrid mechanism. The law should cover cases in which obesity or its sequelae generate substantial and lasting functional limitations or are institutionally treated as such. However, weight discrimination also needs explicit recognition as an autonomous form of exclusion in employment, education, health and services. Without that second pillar, legal systems will continue to offer protection at the cost of requiring people to translate their primary injury (weight exclusion) into the language of disability.

Open questions remain. Direct evidence on the stigmatizing or destigmatizing effect of recognizing obesity as a disability remains limited; subnational heterogeneity, especially in the United States, Canada, and Australia, complicates any strong generalization; and part of the relevant doctrine is still dispersed in administrative decisions and labor courts. However, this uncertainty reinforces the central thesis. Because disease, disability, and weight discrimination are not synonymous, we need an international research and public policy agenda that treats weight-related exclusion as a public health and human rights challenge without denying that severe obesity can, under specific circumstances, generate legitimate disability protection.

## Data Availability

The original contributions presented in the study are included in the article/supplementary material, further inquiries can be directed to the corresponding author.
